# The Physical Fitness Effects of a Week-Long Specialist Tactical Police Selection Course

**DOI:** 10.3390/ijerph17186782

**Published:** 2020-09-17

**Authors:** Ben Schram, Jeremy Robinson, Robin Orr

**Affiliations:** 1 Faculty of Health Science and Medicine, Bond University, Robina 4226, Australia; rorr@bond.edu.au; 2 Tactical Research Unit, Bond University, Robina 4226, Australia; 3 Specialist Response Group—Tactical Response Team, Australian Federal Police, Canberra 2601, Australia; Jeremy.Robinson@afp.gov.au

**Keywords:** police, military, specialist, selection, weight loss, physical performance

## Abstract

Specialist police tactical teams, like special operations military personnel, are tasked with dangerous, high risk missions which are beyond the scope of general police. Consequently, the selection courses for entry into these teams are physiologically and psychologically demanding. The purpose of this study was to examine the physiological effects of a five-day selection course to aid in candidate preparation and course planning. Measures included body mass, grip strength, sit-and-reach flexibility, and a vertical jump assessment. Eleven candidates finished the selection course with significant decreases in body mass (−2.05 kg, *p* = 0.006 (95% CI = 3.65–0.45)), grip strength in the right (−14.48 kg, *p* < 0.001 (95% CI = 21.32–7.64)) and left (−14.27 kg, *p* < 0.001 (95% CI = 21.89–6.66)) hands and in sit-and-reach flexibility (−6.64 cm, *p* < 0.001 (95% CI = 9.94–3.33)). No significant decreases in power output or peak jump velocity of 669.77 W (95% CI = 1942.92–603.39) and 0.28 m/s (95% CI = 0.69–0.14) were found and a non-significant, overall increase in vertical jump height of 6.09 cm (95% CI = −6.08 to 18.79) was seen. Decreases in body mass, grip strength and lower limb flexibility are evident in a grueling five-day selection course. Individuals planning on attending these courses should plan for these negative effects and build redundancy into their performance to minimize the effects of fatigue, decrease injury risk and maximize chances of completion.

## 1. Introduction

Specialist tactical police, like special operations military personnel, are tasked with dangerous, high risk missions which are beyond the scope of general police and military personnel [1]. These high risk tasks may include hostage rescue, execution of high risk warrants, personal protection, or counter terrorism responses [2]. Special operations taskings are known to lead to increases in heart rate, stress, blood lactate, anxiety and dehydration [3]. Members of these elite units are therefore known to possess physiological attributes which exceed that of general police and military personnel [4].

Consequently, the selection courses for entry into these units are physiologically and psychologically demanding, designed to match the operational requirement of this job role [5]. Reported failure rates in military special forces range from 45 to 55% in the US [6] to 30 to 82% in Australia [5] and 69% in Specialist Police in Australia [1]. The length of these courses may range from as little as one week [1] to 10 weeks [3] and they are designed to identify individuals who have the physical capabilities desired within these units [1]. Selection courses typically consist of extreme physical challenges under constant individual and team pressure, often with both sleep and nutritional deprivation [7]. Events conducted during these courses may include stimulus response scenarios with armed offenders, loaded pack marches and repeated stores carries [8]. The physiological consequence of this may include deleterious changes in body composition, strength, power output and subsequent decreases in physical performance [3,9]. Declines in performance and body mass during these courses have been linked to both decreases in muscle mass from nutritional deficits and dehydration from sweating and low fluid intake [3]. Despite some information existing on military specialist selection courses, little is known about the typically shorter, specialist police selection courses.

In order to maximize candidate preparation, reduce the number of failures and better design selection courses, it is important to understand not only the physical requirements of these courses but also the physical effects of participation on these courses. Greater insight into the effects of a course can enable agencies to better plan courses, provide information to potential candidates and ensure injury risk in minimized. The aim of this investigation was to examine the physical fitness effects of a one-week specialist tactical police selection course on candidates attempting selection. It is hypothesized that the intense nature of the selection course will elicit significant declines in physical performance. 

## 2. Materials and Methods 

Prospective data were collected in a non-identifiable form pertaining to various physiological measures for candidates attending a five-day, specialist police tactical team selection course. Over this time-frame, the officers were required to complete 97 serials of which all had different competency and performance measures and required varying intensities and modalities of fitness. All events in the selection process were designed to determine whether the officer was deemed suitable to commence training as a specialist tactical response police officer. All measures were taken at the same time daily, apart from body mass, which was taken in the morning and evening. 

### 2.1. Participants

A total of 18 male candidates (mean age 32.1 ± 5.0 years, mean height 183.7 ± 5.8 cm, mean mass 89.4 ± 8.6 kg) began the selection course which was conducted at an Australian police establishment. Ethical approval was provided by the Bond University Human Research Ethics Committee and gatekeeper approvals were provided by the Australian Federal Police. The following measures were taken.

### 2.2. Body Mass

Body mass was measured (Tanita BC-545N Body Composition Monitor, Arlington Heights, IL, USA) to the nearest 0.01 kg with no additional equipment worn, in physical training gear and without shoes. This process was repeated daily as an estimation of lost body mass through either dehydration or body composition changes. 

### 2.3. Grip Strength

Grip Strength was measured to the nearest 100 g using a handgrip dynamometer (Jamar, Sammons Preston, Bolingbrook, IL, USA). The protocol directed by Dortkamph [10] was followed whereby the handle of the dynamometer was measured from the palm of the hand to the middle of the four fingers. Candidates were instructed to keep their arm by their side without touching the body and elbow fully extended with a neutral grip. On command, they were told to squeeze the dynamometer in a smooth action and maintain the contraction for 5 s. Two trials were taken but if the candidate had a difference of greater than 5% between the 1st and 2nd measurements, then a 3rd trial was undertaken. The highest measure of the two or three attempts was recorded. These measures were repeated at the same time daily as a measure of cumulative fatigue. Grip strength was selected as an outcome measure due to its association as a marker of fatigue [11,12] and association with elements of tactical performance in these populations [13].

### 2.4. Flexibility

Flexibility was measured using a sit-and-reach assessment on a Baseline Sit and Reach Trunk Flexibility Test Box (Baseline Evaluation Instruments, New York, NY, USA). This assessment was conducted as per the protocol by Dortkamph [10]. Candidates were instructed to maintain their feet against the wall of the box and reach forward as far as they could without breaking contact of their fingers with the metal slide. If this occurred, the assessment was redone. Flexibility was measured to the nearest 0.1 cm. 

### 2.5. Power Assessment

Three assessments of power were undertaken including an unloaded jump test, bench throw, and bench pull. Data for these tests were collected using a GymAware Linear Position Transducer Optical Encoder (50-Hz sample period attached to the relevant bar with data smoothing or filtering; Kinetic Performance Technology, Canberra, Australia), connected to an iPad (Version 3, Apple Inc., Cupertino, CA, USA) via Bluetooth connection. Prior to testing, candidates performed a full body dynamic warm up followed by a specific warmup including body weight squats, body weight countermovement jumps (CMJ), pushups, banded pull aparts, clap pushups, inverted TRX rows and medicine ball chest passes. This warmup was progressive in nature from approximately 50% heart rate max to 95% heart rate max. All candidates were familiar with the testing procedure from previous assessments during baseline testing and the assessments were performed at the same time each day in the same location. The specific protocol for each assessment is outlined below. 

#### 2.5.1. Unloaded Jump Test (CMJ)

For assessment of CMJ, a wooden dowel bar was positioned on the upper trapezius/posterior deltoid muscles of the back. The stance was constrained to 15 cm from the lateral side of the candidate’s deltoid as specified by McBride [14]. Candidates initiated the squat via a downward countermovement to a self-selected depth, followed immediately by a vertical jump. Candidates were instructed to keep downward pressure on the wooden dowel bar throughout the jump and encouraged to reach a maximal jump height with each trial in an attempt to maximize power output and velocity. Candidates were encouraged and reminded between trial attempts to jump for “Max height and Max speed”. Each candidate performed 3 trial jumps, with a small pause in between each trial to reset their body in a tall upright position. Candidates were measured for both peak power output (Watts), Velocity m/s and jump height (cm). The CMJ were tested at the same time daily as a measure of lower body fatigue.

#### 2.5.2. Bench Throw

For the bench throw assessment, candidates lay supine on a flat bench with arms extended straight over the shoulders. Candidates unhooked a barbell that was attached to a Smith machine from the safety slots and lowered it to the chest area. The stops of the Smith machine were placed in a position whereby the bar rested upon them at a height of about 10–15 cm from the chest of the candidate. This was the start position from where the concentric movement of the bench throw exercise was initiated in a vertical plane. An approximate 1-s pause was implemented between repetitions to negate any significant usage of elastic energy from the stretch shortening cycle (SSC) contributing to the power output and velocity result. The candidates were instructed to initiate the movement by throwing the bar for maximal height at the end of the concentric movement.

The candidates then caught the bar on its descent and positioned the bar back on the Smith machine stops for 1 s before commencing the remaining repetitions. Three repetitions were performed in each set with an absolute weight of 40 kg attached to the barbell as a study by Drinkwater and colleagues [15] investigated the reliability of mean power in a 40 kg bench throw with high levels relative reliability found. The physical performance variables measured and used for analysis were peak power (W) and peak velocity (m/s).

#### 2.5.3. Prone Bench Pull Concentric

For the assessment of the prone bench pull, candidates were instructed to lay in a prone position on a bench frame with chin down, arms extended to the outside of the bench just outside shoulder width with a prone overhand grip on the barbell. From that position the candidates were instructed to pull the barbell loaded with 2 × 20 kg rubber coated Australian Barbell Company plates (60 kg absolute weight) as hard and fast as possible until contact was made with the barbell on the underside of the metal bench. The legs were not held, and the chin remained in contact with the bench at all times. 

A total of three repetitions were performed in total with a one second pause at the bottom between repetitions. This was in the start position with the arms fully extended. The physical performance variables measured and used for analysis were mean power (W), peak power (W), mean velocity (m/s) and peak velocity (m/s). 

*Statistical Analyses:*

Descriptive statistics including means, standard deviations, mean change and 95% confidence intervals were calculated on the Statistical Package for the Social Sciences (SPSS, Version 24, IBM). Repeated measures ANOVAs were calculated to determine differences between measurements with a Bonferroni adjustment for multiple comparisons performed post hoc. Sphericity was checked in all measurements and where violated, Greenhouse–Geisser adjustments were made. Alpha levels were set at 0.05 a priori. Effect sizes were calculated with a partial eta squared (ƞ^2^) for each ANOVA. 

## 3. Results

From an initial 18 candidates, 11 completed the selection week with two self-withdrawing and five medically withdrawn. Demographics and initial measures of those who did and did not complete the selection course can be seen in Table 1. Despite those who completed the week on average being younger and shorter than those who did not complete the week, the differences were not significant. The only significant difference in initial measures between those who did and did not complete the selection course was body mass, with those who completed the course being significantly lighter than those who did not complete the course (F = 0.042, t = 2.934, *p* = 0.010). 

### 3.1. Body Mass

**Figure 1:**
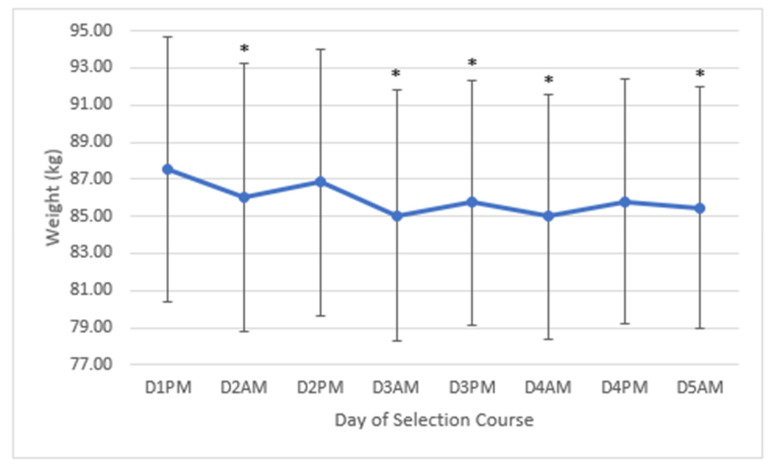
The group average changes in body mass (±SD) over the course of five days of morning (AM) and afternoon (PM) measurements. * = Significantly less than day one (*p* < 0.05).

Over the course of the week, the average loss in mass from the candidates who did complete the week was 2.05 kg (95% CI = 3.65–0.45) or around 2.4% of their starting body mass. Candidates had a significant decrease in their body mass by the second measurement on the morning on day two of 1.48 kg (95% CI = 2.45–0.51) (F(7,70) = 16.572, *p* = 0.002, partial ƞ^2^ = 0.62). This decline in body mass can be seen graphically in Figure 1.

### 3.2. Grip Strength

**Figure 2:**
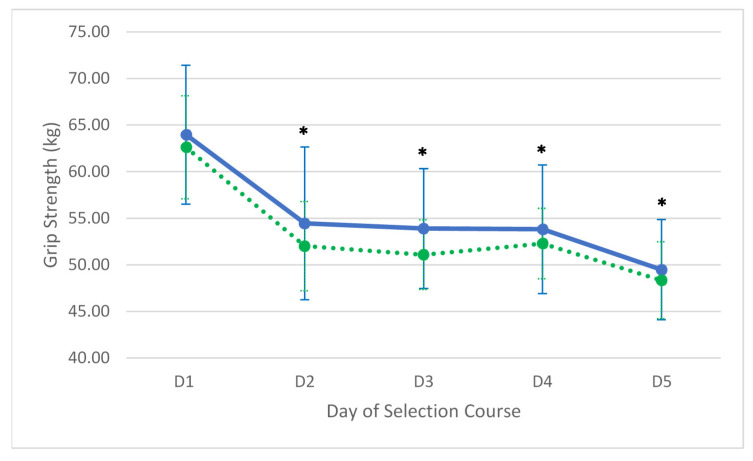
Average changes (±SD) in grip strength over the five days. The solid line is the right hand and the dotted line is the left hand. * = Significantly less than day one (*p* < 0.05).

Grip strength declined in total by 14.48 kg (95% CI = −21.32 to −7.64) on the right and 14.27 kg (95% CI = −21.89 to −6.66 kg) on the left. The total decline on both the right (F(4,40) = 27.785, *p* < 0.001, partial ƞ^2^ = 0.74) and left (F(4,40) = 23.178, *p* = 0.001, partial ƞ^2^ = 0.70) hands was significant. Grip strength on the right (−9.52 kg (95% CI = −14.10 to −4.91 kg)) and left (−10.61 kg (95% CI = −16.67 to −4.55 kg)) had decreased significantly by the second day of the course. The decline in right and left-handed grip strength can be seen graphically in Figure 2.

### 3.3. Flexibility

**Figure 3:**
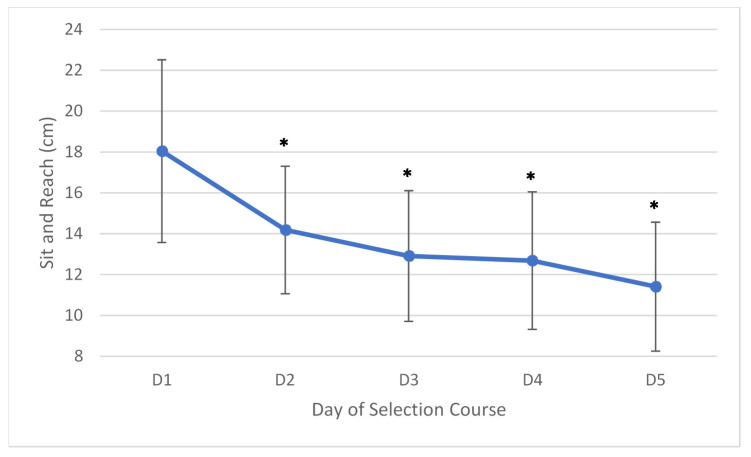
Average changes (±SD) in sit-and-reach flexibility over the five days. * = Significantly less than day one (*p* < 0.05).

A significant (F(1.431,14.310) = 33.550, *p* < 0.001, partial ƞ2 = 0.77) loss in sit-and-reach flexibility of 6.64 cm (95% CI =9.94–3.33) was found over the duration of the course. Sit-and-reach flexibility had already declined significantly by 3.86 cm (95% CI = 6.67–1.06) by the second day (F(4,40) = 33.550, *p* = 0.006). A graphical representation of this decline can be seen in Figure 3 below.

### 3.4. Lower Limb Power Output

**Figure 4:**
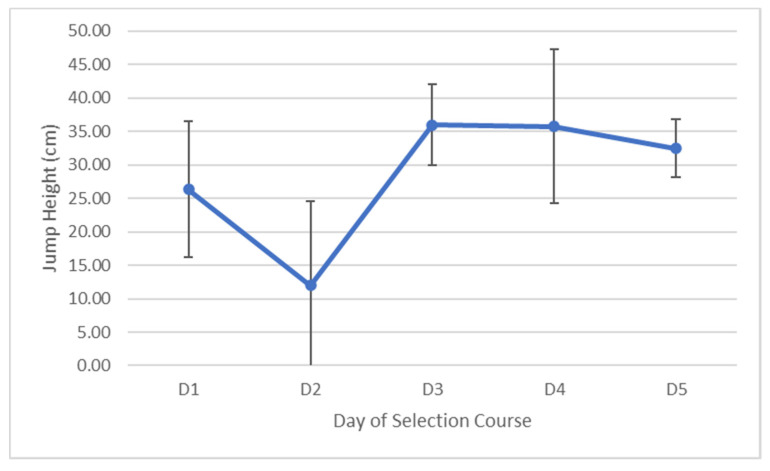
Average changes (±SD) in vertical jump height as measured by a vertical jump assessment over the five days.

**Figure 5:**
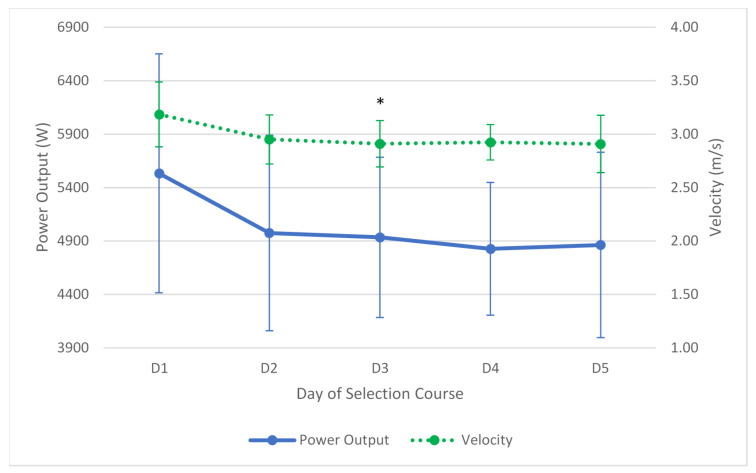
Average changes (±SD) in vertical jump performance over the five days. Solid line represents power output, dotted line represents velocity. * = Significant decrease in jump velocity compared to day one (*p* < 0.05).

Jump height as measured by a vertical jump assessment was seen to increase on the final day in seven of the eleven candidates who finished the week. Despite an overall increase in jump height of 6.09 cm (95% CI = −4.47–18.78), the difference between the initial measure and final measure was not significant (F(1.942,19.422) = 14.999, *p* = 1.000, partial ƞ^2^ = 0.63). Despite three candidates improving their initial power output on the vertical jump on the final day of the selection course, the group on average decreased by 669.77 W (95% CI = 1942.92–603.39), which despite large confidence intervals, was not a significant decline (F(4,40) = 2.654, *p* = 0.889, partial ƞ^2^ = 0.21). Over the entire course, jump velocity declined by 0.276 m/s (−0.69 to 0.14 m/s) which was also not a significant decline (F(2.145,21.448) = 4.426, *p* = 0.392, partial ƞ^2^ = 0.31). The jump velocity on the third day was significantly (F(2.145,21.448) = 4.426, *p* = 0.027) lower than the first day by 0.275 m/s (95% CI = 0.52–0.03). The changes in vertical jump height, power output and jump velocity are seen in Figure 4 and Figure 5.

### 3.5. Upper Limb Power Output

**Figure 6:**
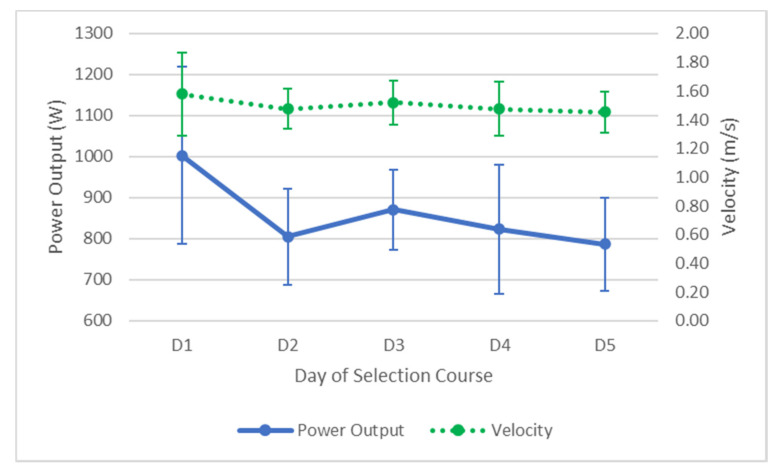
Average changes (±SD) in bench throw performance over the five days. Solid line represents power output, dotted line represents velocity.

There was a 216.72 W (95% CI = 471.06–37.61) decline in bench throw power on average over the duration of the selection course, however this difference was not significant (F(4,40) = 7.041, *p* = 0.122, partial ƞ^2^ = 0.41). There was also a decline in peak throw velocity of 0.12 m/s (95% CI = 0.47–0.22); however, this decline was not statistically significant (F(1.86, 18.595) = 1.235, *p* = 1.000, partial ƞ^2^ = 0.11). A visual representation of this decline is seen in Figure 6.

### 3.6. Bench Pull

**Figure 7:**
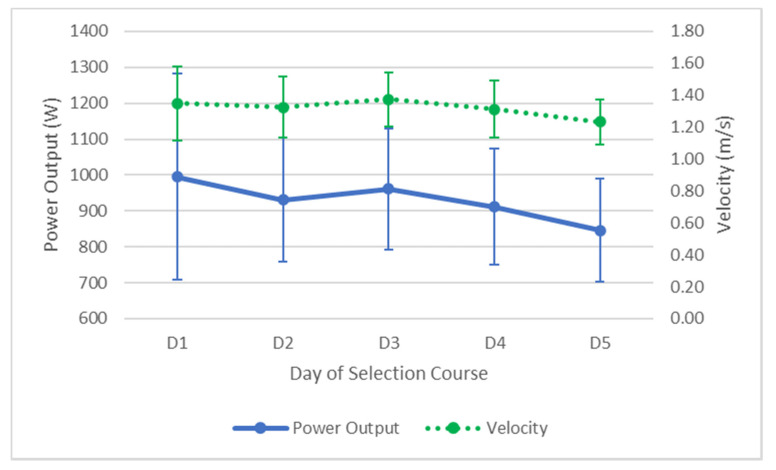
Average changes (±SD) in bench pull performance over the five days. Solid line represents power output, dotted line represents velocity.

Power output on the bench pull decreased by 148.43 W (95% CI = 357.34–60.49) which was not a significant difference (F(2.159, 21.595) = 3.333, *p* = 0.291, partial ƞ^2^ = 0.25). Peak velocity of the bench pull exercise also decreased over the selection course by 0.12 m/s (95% CI = 0.29–0.06), however this difference was not significant (F(4,40) = 3.448, *p* = 0.377, partial ƞ^2^ = 0.26). This decline is represented graphically below in Figure 7.

## 4. Discussion

The aim of this study was to examine the physical fitness effects of a one-week specialist police selection course on candidates attempting selection. Key findings were that significant decreases were observed over the duration of the course in many key variables. The physiological declines found in this study are similar to previous research in special operations soldiers attending military special operations selection courses [3], U.S. Army Ranger Training courses [9], and overseas deployments [16]. In line with other research, no one single measure was associated with success on this course [17]. Specialist police need high levels of fitness, strength and power along with other factors not measured in this study including mental resilience and the ability to operate under conditions of sleep and nutritional deprivation.

There were significant losses in body mass over the duration of the course of 2.05 ± 1.26 kg or around 2.4% of participants body mass. Despite minimal research done in this area for comparison, this loss in only five days is greater than what has been reported from a much longer 9-month deployment in Afghanistan of 1.9% [16]. It is, however, less than the 3.1% decrease reported in the final four-day exercise of a special forces selection course [3] and much less than reports of 13% during 8 weeks of US Army Rangers Training [9]. Despite measuring overall body mass due to it not being feasible to measure body composition at consistent intervals throughout the course, it remains difficult to determine the exact ratio of water, muscle and fat which was lost over the duration of the course. Hormeno-Holgado et al. [3] demonstrated that there are variations in the ratios of muscle mass and fat lost during selection courses, with those with more of a sympathetic response with greater heart rates hypothesized to have lost more muscle mass as opposed to fat mass.

Declines in grip strength over the course of the week are thought to be due to central nervous system fatigue, sleep deprivation, decreased physiological function due to dehydration, energy deficits or a combination of both [3]. Despite showing a 3.8% increase in grip strength after a four-day exercise on a special force’s selection course, it should be noted the study by Hormeno-Holgado et al. [3] measured grip strength at the end of the exercise. Sadness, alertness and tension were found to decrease immediately at the completion of the exercise, which may have explained the increase in grip strength. As a comparison, the grip strength measures were taken in the morning on the final day of this study while still being under the direction of selection staff, which may have negated the sense of accomplishment for these candidates. Under conditions of fatigue, physiological and cognitive decline is evident [18] and grip strength has been found to be important for occupational specific tasks in policing, most notably marksmanship and tactical options assessments (TACOPS) [19]. Candidates who are in a state of fatigue may therefore perform poorly in occupationally relevant tasks at latter stages of a specialist selection course simply due to the cumulative fatigue associated with the course.

Flexibility, as measured by the sit-and-reach test, was seen to decline significantly over the course of the selection week. While the absolute value of the sit-and-reach test as a measure of hamstring flexibility may not be associated with injury risk [20], it may be more representative of a decline in lower limb flexibility and joint stiffness more globally, which may be of note. Increases in joint stiffness which have been shown to be caused by load carriage, are thought to transfer up the kinetic chain to other joints [21]. Not only may this predispose candidates to injury in the latter stages of a selection course [22], it may also highlight an injury risk for those who are already in the specialist police role who are working at a high operational tempo.

There were decreases in both power output and jump velocity after the completion of the selection course, these were not deemed to be significant. Interestingly and unexpectedly, vertical jump increased by 23.1% over the selection course, potentially due to an increase in passive stiffness, and decrease in active stiffness in the lower limb and consequent increased ability to store elastic potential energy [23]. Evidence of this ability to redirect energy through the hysteresis loop may be highlighted by the increases in joint stiffness as measured by the sit-and-reach assessment. The decrease in active stiffness may be due to neuromuscular fatigue, a transient effect of the course similar to the decrease in active stiffness and subsequent power output associated with ageing [24]. Another explanation might simply be that the first measurement was influenced by the heightened levels of stress and arousal of candidates on the first day of the course, and that their performance normalized after becoming accustomed to the tempo and demands of the course. The subsequent measures of around 35 cm are in line with previously reported heights amongst Marine Infantry Officer Course candidates [25].

There was a 12% decrease in power output in the vertical jump in this study, similar to the decrease found after a period of US Army Ranger Training of 21% [9]. In contrast to the results of this study, a concurrent decrease in vertical jump height of 16% was found in those soldiers completing Ranger Training [9]. Four days of special forces selection course exercises led to a 12.5% horizontal jump distance decline in the study by Hormeno-Holgado et al., despite it being measured at the completion of the exercise [3]. Declines in vertical jump height after a much longer, nine-month deployment have been reported as just 1% [16]. While decreases in vertical jump height have been linked with a significantly greater risk of both injury and illness in police personnel [26]. Sustained operations in US Marines have been shown to lead to declines of 4.9% in countermovement jump height (m) and 8.9% in power (W), both of which affected overall performance [27]. The large decrease in vertical jump measures in the second day of this study are thought to be due to the timetabling of the selection course. Candidates were tasked with both a load carriage event and another task with a high number of stairs on the first night, which would have elicited high levels of neuromuscular fatigue [28] leading to decreases in both physical and cognitive performance [29,30]. Additionally, a greater amount of rest was given after the first night, which may also have had some contribution to the relative improvements in performance in the latter stages of the week.

There were minimal changes in power output in the upper limb as measured by both the bench throw and also the bench pull. It would appear as though the lower limb is much more affected by fatigue than the upper limb, possibly due to the prolonged load carriage and tasks which predominately use muscles of the lower limb. The lower limb is also more commonly injured than the upper limb in these sorts of tactical populations [31].

Overall, successful candidates were generally younger and shorter but not significantly lighter than unsuccessful candidates. Through the course, over a period of one week, declines were seen in body mass, grip strength, flexibility, and power output. Given that there are documented declines in these measures, strength and conditioning coaches who are working with candidates should ensure they train strength, flexibility and power for as much redundancy as possible and where able, expose themselves to periods of sleep and nutritional deficit and understand how their performance is affected under prolonged periods of stress. Likewise, agencies should be aware than when they put candidates under the required level of fatigue, sleep and nutritional deprivation, they may be concurrently increasing injury risk. Agencies should plan for adequate breaks post selection course for recovery of these parameters before launching into any reinforcement or tactical skills validation cycles of continuing training.

The small sample size available is a clear limitation of this study. It should be noted, however, that data from the entire selection course were used and other studies in this area have also used smaller sample sizes, given the unique nature of this occupation [1,5]. The measure of body composition would have been ideal, however given the logistical and time burden to perform these measurements, it was not feasible to conduct this measurement. Future studies should attempt to measure composition changes to determine the exact nature of this decline in body mass.

## 5. Conclusions

Decreases in body mass, grip strength, lower limb flexibility and lower limb power output are evident following a grueling five-day specialist selection course, which may be associated with an increased injury risk. Individuals planning on attending these selection courses should plan for these negative effects and build redundancy to minimize their effects in an attempt to decrease injury and maximize the chances of completion.

## Figures and Tables

**Table 1:** Comparison of candidates who completed and did complete the selection course. Data presented as mean SD. * = Significant difference (*p* < 0.05).

	Completed (n = 11)	Not Completed (n = 7)
Age (years)	30.64 ± 4.97	34.43 ± 4.51
Height (cm)	182.82 ± 4.85	185.14 ± 7.20
Body Mass (kg)	87.52 ± 7.15 *	98.10 ± 7.95
Grip Strength Right (kg)	63.96 ± 7.46	59.12 ± 6.20
Grip Strength Left (kg)	62.62 ± 5.53	58.96 ± 6.71
Sit and Reach (cm)	18.05 ± 4.47	19.07 ± 5.48
Vertical Jump Height (cm)	26.36 ± 10.22	28.57 ± 7.28
Vertical Jump Power (W)	5532.94 ± 1117.35	5553.16 ± 760.05
Vertical Jump Velocity (m/s)	3.18 ± 0.30	3.02 ± 0.14
Bench Throw Power (W)	1003.20 ± 215.34	999.45 ± 162.92
Bench Throw Velocity (m/s)	1.58 ± 0.29	1.56 ± 0.24
Bench Pull Power (W)	994.55 ± 286.76	1087.54 ± 345.44
Bench Pull Velocity (m/s)	1.35 ± 0.23	1.41 ± 0.26
